# Toward Computational Motivation for Multi-Agent Systems and Swarms

**DOI:** 10.3389/frobt.2018.00134

**Published:** 2018-12-18

**Authors:** Md Mohiuddin Khan, Kathryn Kasmarik, Michael Barlow

**Affiliations:** School of Engineering and Information Technology, University of New South Wales, Canberra, ACT, Australia

**Keywords:** intrinsic motivation, artificial intelligence, cognitive development, swarms, multi-agent systems, exploration, curiosity

## Abstract

Motivation is a crucial part of animal and human mental development, fostering competence, autonomy, and open-ended development. Motivational constructs have proved to be an integral part of explaining human and animal behavior. Computer scientists have proposed various computational models of motivation for artificial agents, with the aim of building artificial agents capable of autonomous goal generation. Multi-agent systems and swarm intelligence are natural extensions to the individual agent setting. However, there are only a few works that focus on motivation theories in multi-agent or swarm settings. In this study, we review current computational models of motivation settings, mechanisms, functions and evaluation methods and discuss how we can produce systems with new kinds of functions not possible using individual agents. We describe in detail this open area of research and the major research challenges it holds.

## Introduction

Artificial intelligence has come a long way toward developing intelligent systems. We are in an era where autonomous cars are on the verge of roaming the streets, chess programs can beat grandmasters, and handheld devices understand and translate speech in real-time. However, we are still far from developing artificial agents capable of demonstrating, human-like adaptive behavior, and open-ended learning. Research areas such as autonomous mental development (Thomaz and Breazeal, 2006) and developmental cognitive robotics (Asada et al., 2009) aim to address these challenges.

One of the important features of the artificial agents of the future will be their ability to gain knowledge and learn skills without explicit feedback from humans as well as adapt the behavior according to external and internal needs. As described in Russell and Norvig (2016), agent behavior can be guided by goals and utilities. In this paper we define motivation as a mechanism that generates goals as an intermediary between sensation and action selection in an agent. These “motivated” agents will use their acquired knowledge and skills to build increasingly complex repertoires of behaviors. Computational models of motivations have been proposed, drawing inspiration and insight from biological (Gatsoulis and Mcginnity, 2015), neural (Gottlieb et al., 2016), and evolutionary (Singh et al., 2010) perspectives. Computational models of motivation enables artificial agents to gather knowledge, seek competence, and select goals based on a combination of their individual experiences, preferences, and environmental characteristics (Merrick and Shafi, 2011). While the general concept of motivation is broad and has many facets, in our paper we focus on how motivation has been defined in artificial agents. Essentially a human and animal trait, researchers have used various notions, mathematical models, and frameworks to define motivation for artificial agents.

Inspired by insights from human psychology, these motivational models propose to incorporate open-ended learning, autonomous skill acquisition, and progressive learning in artificial agents. However, the current computational models of motivation have seldom been extended and explored in the social context- where multiple artificial agents exist, communicate and interact. Multi-agent systems and swarms are two examples of such contexts.

Multi-agent systems are a logical extension of the single agent idea. Studies involving multi-agent systems include the communication and behavior (Panait and Luke, 2005) among multiple artificial agents. With combined goals, actions, domain knowledge, and interactions (Stone and Veloso, 2000), these systems pose unique challenges that are absent in single agent settings. They can be robust and scalable, while introducing complexity in cooperation and communication, and capable of providing a platform to build social intelligence for artificial agents (Dautenhahn, 1995).

The term “Swarm intelligence” was first coined by Beni and Wang (1993). As they explained (Beni, 2004), a machine can be defined as “intelligent” if it demonstrates behavior which is “*neither random nor predictable*.” Following this definition, an intelligent swarm was defined as a group of non-intelligent agents that can collectively demonstrate intelligent behavior. Swarm intelligence is inspired by behavior occurring in insects, birds, fish and other organisms in nature. These biological systems have existed for millennia, and they have been efficiently solving complex problems through apparently simple rules. The current approach to swarm intelligence is a culmination of observations and findings from both biologists (Beekman et al., 2008) and computer scientists (Brambilla et al., 2013).

In this paper, we review the state of the art of the computational models of motivation and present a comprehensive review of this research. We talk about the various aspects of the current works and discuss the scope of extending them to multi-agent systems and swarms. This article has the following main contributions:

1. We review existing computational models of motivation, based on their setting, mechanism, function, and evaluation methods. This provides a structured overview of the existing research and a framework in which to introduce multi-agent systems and swarms to the study of computational motivation.
2. We characterize the open area of research that involves computational models of motivation in a social context, specifically, multi-agent systems, and swarms. We do this by examining motivation on the traditional axes of competence and knowledge, while introducing a new axis of social vs. individual agents.
3. Finally we determine the major challenges and benefits of designing computational motivation for multi-agent and swarm settings.

In the related works section, we discuss the existing surveys on computational models of motivation and justify the position of this survey. In the next section, we present the main contribution of this paper. It starts with a discussion on motivation and intrinsic motivation (IM), then provides a structural summary of the current works on computational models of intrinsic motivation. It concludes by presenting an outlook and summary on computational motivation in a social context. In the discussion section, we discuss the social side of intrinsic motivation from various aspects, present the major research challenges in that context, and conclude the paper.

## Related Work

A number of surveys have been produced in the area of computational motivation. This section discusses the focus of each of the existing surveys and justifies the need for a new survey studying computational motivation in a social context.

Oudeyer and Kaplan (2007) provided a typology of computational approaches to motivation. They assumed that any particular Computational Reinforcement Learning (CRL) framework could be used to realize motivation signals. Hence, their typology is based on the formal definition of the reward used in a framework. Characterizing a robot by having a number of sensor channels and motor channels, they classified motivation models into the following categories:

1. Knowledge-based: Knowledge-based systems use “measures that are related to the capacity of the system to model its environment” (Mirolli and Baldassarre, 2013b). The knowledge, for example, can be computed from the past sensorimotor values. Once the knowledge is acquired, the difference between the estimated knowledge and the actual perceived value can be used to design the reward. For example, the intrinsic reward can be proportional to the improvement of the prediction. In this case, the robot will be “intrinsically motivated” to maximize prediction progress, i.e., to minimize the prediction errors. In essence, knowledge-based models put the emphasis on how much an artificial agent “knows” about the environment.
2. Competence-based: In these models, the reward for intrinsic motivations is designed based on what an agent “can do” with respect to a particular goal or task. In these models, intrinsic rewards are associated with an agent's ability to reach a certain state or perform a certain activity. For example, a robot can be rewarded proportionally to the progress in learning a task, driving it toward goals that are rapidly improvable and deterring it from situations that are too difficult or too easy to gain enough competence.

In the survey section, we will use these categories as the baseline from which we introduce motivation in a social context. A related review (Oudeyer et al., 2007) describes a robot as having two modules—a learning machine and meta-learning machine. While the learning machine learns to predict the sensorimotor consequences of an executed action, the meta-learning machine learns to predict the errors of the learning machine. The prediction made by the meta-predictor is used as an intrinsic reward. The authors divided the existing approaches in three categories based on how these predictions are exploited to generate intrinsic motives. These are error maximization (Thrun, 1995; Huang and Weng, 2002; Barto et al., 2004; Marshall et al., 2004), progress maximization (Herrmann et al., 2000; Kaplan and Oudeyer, 2003), and similarity-based progress maximization (Schmidhuber, 1991). This survey provides an effective classification of the computational motivation models by distinguishing between learning and meta-learning with a focus on how agents can explore environments to gather effective information.

While the previously mentioned surveys discussed the mechanisms of intrinsic motivation, they do not provide a clear insight on the functional roles of motivation. (Mirolli and Baldassarre, 2013a,b) focus on the knowledge-based and competence-based models of intrinsic motivation from the functional aspect. They argued that “*the ultimate function of intrinsic motivation is to support the cumulative learning of skills rather than knowledge*.” They have analyzed some of the knowledge-based mechanism regarding their contribution to competence acquisition. They argue that to facilitate cumulative skill acquisition based on intrinsic motivation, one has to focus on the hierarchy and modularity of the skill organization framework. In this framework, knowledge-based reward signals can act at a lower level to learn the world-model, while competence-based signals can work as a selector deciding which skill is to learn.

Schmidhuber (2010) proposed a typology of intrinsic motivation from a different perspective. He pointed out that most of the intrinsically motivated systems have the following components:

1. A model/encoder/predictor that captures the sensory inputs, internal states, reinforcement signals and actions.
2. An intrinsic reward scheme that determines the learning progress of the model.
3. A reinforcement learner that maximizes the future expected reward.

Hence, this typology can be created considering the types of these components. A set of companion questions for each of the components is added and answering the questions can help build a detailed topology. Schmidhuber presented a general, theoretical framework that one can use to build a typology of intrinsically motivated systems. This typology is based on the consideration that intrinsic motivation will always be implemented through reinforcement signals.

Barto (2013) provides an overview of intrinsic motivation with regard to Reinforcement Learning (RL). He highlighted the suitability of RL to capture the principles of motivation in artificial systems by connecting drive theory with reward maximization. Barto pointed out that RL framework “*does not care*” where the reward signal is coming from. This makes it possible to introduce an “intrinsic reward” which would be generated from within the organism but won't affect the whole RL mechanism. Hence it can naturally accommodate intrinsic motivation. In addition to that, he highlighted that intrinsic reward signals can mimic the evolutionary success of organisms.

Computational models of motivations were surveyed as a part of computational value systems (Merrick, 2017). Value systems define behavioral responses of intelligent beings with regard to the external environment. The term “computational value systems” extends the idea toward artificial agents such as robots. A brief summary on the implementation of motivated reinforcement learning as value systems is provided. Merrick (Merrick, 2013) further reviews the existing novelty-based models of intrinsic motivation with a focus on building combined motivation models and integrated learning architecture.

In a more recent work (Roohi et al., 2018), application of intrinsic motivation in player modeling and game testing was reviewed. This work concludes that while a few parameters of intrinsic motivation are frequently implemented in the existing works, some important features are generally overlooked. They also point out the need for more complex motivational models and better ways to evaluate them. The purpose of our paper is to extend the existing views on intrinsic motivation beyond individual agents to multi-agent and swarm settings.

A summary of the existing surveys is presented in Table 1. It is evident from the table that there is no recent survey on computational intrinsic motivation that provides insight into its use in a social context. Till now, the most commonly referred to survey of intrinsic motivation is the typology provided by Oudeyer and Kaplan (2007). Their seminal work provides a comprehensive view of a formal framework for motivation. It provides a review of the then existing computational models of IM. Since their work in 2007, intrinsic motivation has become one of the most attractive research areas in cognitive science and autonomous mental development. Moreover, with the emergence of areas such as deep learning (Sigaud and Droniou, 2016; Wong, 2016) on autonomous mental development (Lake et al., 2017), computational models of motivation have become relevant in newer dimensions. In this survey, we categorize the existing approaches and argue the benefits of extending them to social settings- specifically multi-agent or swarm settings.

**Table 1:** Summary of reviews on intrinsic motivation in artificial agents and contribution of our survey.

**Survey**	**Comment**
Oudeyer and Kaplan, 2007	Provides typology based on motivation theory
Oudeyer et al., 2007	Categorizes existing approaches based on learning and meta-learning
	Highlights difference between knowledge-based vs. competence-based motivation models
Schmidhuber, 2010	Provides typology based on reward theory
Barto, 2013	Discusses intrinsic motivation with relation to reinforcement learning
Merrick, 2017	Intrinsic motivation is surveyed as a part of computational value systems
Roohi et al., 2018	Intrinsic motivation is surveyed as a tool to build player models in computer games
In this study	Introduces computational motivation in a social context

## Survey: From Motivation in Individual Agents to Motivation in Social Settings

In the Motivation and Intrinsic Motivation from a Psychological Perspective section, we introduce motivation from a psychological perspective. The next section focuses specifically on intrinsic motivation, as it has been used in computational settings. In the Computational Motivation in Swarm and Multi-Agent Settings section, we survey the current approaches into multi-agent and swarm settings and discuss the possible extensions.

### Motivation and Intrinsic Motivation From a Psychological Perspective

Ryan and Deci (2000a) succinctly defined motivation “*to be motivated means to be moved to do something*.” Motivation is the mechanism that makes humans and animals commit various tasks. Motivation shapes our behavior throughout our life. On the simplest terms, motivation may seem like a straightforward tool that helps an organism to survive. However, a little deliberation can show us the depth, variety and effect of motivation on a grander scale. For example, motivations vary in degree and in type for different people. In a classroom situation, two students doing their homework can be motivated by completely different influences. One of the students may finish the homework for getting high scores in tests, and the other may do it as she finds the subject highly intriguing. Furthermore, a third student may be influence by a combination of both of these motivations, along with a multitude of others.

Researchers from various fields have tried to explain motivation from their respective view. A plethora of concepts on the definition, function, and characteristics of motivation is provided by ethologists (Epstein, 1982), psychologists (Ryan and Deci, 2000a) neuroscientists (Watts and Swanson, 2002; Daw and Shohamy, 2008), and behavioral neuroscientists (Berridge, 2004), among others. The psychological perspective is particularly relevant to our survey. We only provide a brief overview of the psychological theories here. A comprehensive review can be found in Savage (2000).

One of the most influential theories of motivation in psychology is the drive concept, most productively formulated by Hull (1951, 1952). The drive theory states that behavior of an organism is motivated by drives such as hunger and thirst. These drives arise as a response to reduce physiological needs and they motivate behavior which results in the necessary deficit. The theory of drive is centered on the concept of “homeostasis” (Cannon, 1932), where “*bodily conditions are maintained in approximate equilibrium despite external perturbations*.” This theory, which says motivated behavior is a response to encounter changes in equilibrium condition, has influenced many other theories on motivation. Moreover, as described by Savage (Savage, 2000), the theory of drive is an attractive option to model motivational systems for artificial agents. The reason for this is the reduction of drive can be translated to a system's reward mechanism which monitors different variables and respond appropriately (i.e., reduce the deficit in the particular need) when they change. However, because drive theory only explains a subset of animal and human behaviors, other theories have been proposed.

Another motivational theory (Toates, 1986), defines motivation as a multiplicative combination of internal state and an incentive factor. According to this, motivation in an organism will arise as an interaction between an internal state of the organism (e.g., thirst) and an external incentive factor (availability of drinking water). Other motivational theories include the hedonic theories, which state we seek pleasurable activities and keep away from the unpleasant ones.

The shortcomings of the drive theory of motivation become evident if we consider certain human and animal behavior. Human infants perform activities that are not driven toward reducing needs such as hunger or thirst. In one experiment (Harlow et al., 1950), a group of monkeys spontaneously attempted to solve a complex puzzle without any specific reward. Likewise, humans perform various activities such as painting, traveling, and playing sports, which do not directly bring any obvious external rewards. This kind of curious and exploratory behavior are not well-modeled by the drive theory of motivation. White (1959) and Berlyne (1960, 1966) pointed out the abundance of such intrinsically rewarding activities that are driven by curiosity, play and exploration and in absence of explicit reward. This is where the notion of intrinsic motivation gets introduced. Intrinsic motivation is defined as the mechanism that encourages organisms to perform an activity “*for the inherent satisfaction rather than some separable consequence*” (Deci and Ryan, 1985). Intrinsically motivated activities are conducted for the fun and challenge rather than achieving external rewards.

As soon as we introduce the notion of intrinsic motivation, the next question is- “what are the factors that make an activity intrinsically motivating?” Psychologists have proposed quite a few theories in this context. Influenced by drive theory, some psychologists proposed these activities are caused by “drive for exploration” (Montgomery, 1954) and “drive to manipulate” (Harlow et al., 1950). However, as criticized by White (1959), these approaches have shortcomings. Indeed, these exploratory activities are not homeostatic, in contrast to what drive theory has proposed. An alternative stream of the idea to explain intrinsically motivated activities is that of “optimal level theories”. Dörner and Güss (2013) conducted an experiment that involved rats going through various stimulating activities. The experiment provided some key ideas toward intrinsic motivation. It was observed that if animals continue getting a certain level of environmental complexity, they become used to it and eventually gets bored. If they are provided with a slightly complex stimulus, they become curious again. However, if they encounter a stimulus which is too complex compared to their current situation, it confuses them and they tend to avoid it. In effect, an animal will be intrinsically motivated by the activities and stimuli that are optimally difficult and sit in the middle between familiarity and extreme novelty. Berlyne (1960) explored similar notions. As pointed out by Barto (2013), optimal level theories have important applications in varied areas such child development, architecture, city planning, music, and so on.

Deci and Ryan (1985) further presented the Cognitive Evaluation Theory (CET). CET states that intrinsically motivating activities are the ones that satisfy innate psychological needs such as competence, autonomy and relatedness. In classroom situations, factors such as competence, autonomy and self-determination facilitate intrinsic motivation whereas threats, deadlines, competitions, and tangible rewards diminish intrinsic motivation (Ryan and Deci, 2000a). Berlyne (1966) suggested novelty, incongruity, surprise and complexity as underlying factors that affect intrinsic motivation. As we will see in the latter part of this paper, these factors are extensively used in modeling computational intrinsic motivation.

We have detailed the psychological theories behind intrinsically motivated activities and their distinction from extrinsic motivations. A relevant question after this discussion can be- what are the effectual differences between intrinsic and extrinsic motivation and why would one be interested in intrinsic motivation? One answer to this was provided by Baldassarre (2011). He argued that from an evolutionary perspective, extrinsic motivations guide learning of skill, knowledge and behavior which directly increases the “fitness” (defined as survival and reproductive chances) of an individual, whereas intrinsic motivation produces behavior that increase the fitness only at a later stage. We believe this aspect makes intrinsic motivation more complex and interesting. If we want to build human-like artificial intelligent systems, we need to implement mechanisms inducing intrinsic motives and learn skills and knowledge that may not seem useful now. This is where we need to connect the psychological concepts with the computational models.

### Structuring Existing Approaches to Computational Motivation

**Figure 1:**
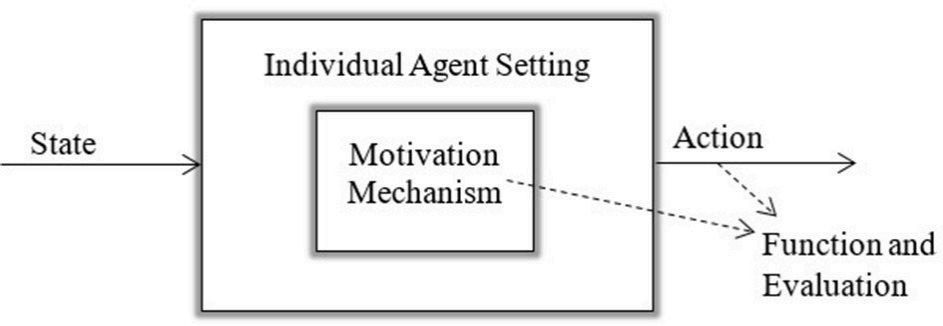
Visualization of the relationship between Setting, Mechanism, Function, and Evaluation. The setting is the artificial intelligence framework wrapped around the motivation mechanism. The motivation mechanism influences the action of the agent, which together produces a measurable function.

In this section, we provide a review of the current computational models of motivation from four different aspects: setting, mechanism, function and evaluation. We base our discussion on the concept of an agent which can sense the state of its world, reason about this state and act. While the motivation mechanism, i.e., how motivation is defined, is central to the broader idea, the peripheral concepts are quite relevant as well. Figure 1 illustrates these concepts, which are further defined in the sections below. The significance of each of these aspects is described in the relevant section, followed by the salient features in the existing work.

#### Setting

The first aspect we examine is the settings in which computational models of motivation are used. By setting, we mean the artificial intelligence framework into which computational motivation has been embedded. Examples include learning and planning settings. In Table 2, we list a cross-section of the areas where computational models of motivation are used. The rows of the table are not mutually exclusive—a single reference can be present in multiple rows.

**Table 2:** Settings in which computational models of motivation are used.

**Settings**	**References**
Reinforcement Learning	Barto et al., 2004; Simşek and Barto, 2006; Schembri et al., 2007; Sequeira et al., 2011; Kompella et al., 2012; Baldassarre and Mirolli, 2013; Metzen and Kirchner, 2013; Di Nocera et al., 2014; Frank et al., 2015; Hester and Stone, 2015
Deep Learning	Mohamed and Rezende, 2015; Kulkarni et al., 2016; Achiam and Sastry, 2017; Zhelo et al., 2018
Hierarchical Structure	Schembri et al., 2007; Baranes and Oudeyer, 2010; Baldassarre and Mirolli, 2013; Santucci et al., 2013; Frank et al., 2015; Kulkarni et al., 2016
Active Learning	Oudeyer et al., 2007; Baranes and Oudeyer, 2009, 2010, 2013; Kompella et al., 2017; Pathak et al., 2017
Motion Planning	Frank et al., 2015
Affordance Discovery	Hart et al., 2008; Hart, 2009
Goal Discovery/Goal Generation	Salgado et al., 2016; Santucci et al., 2016; Kompella et al., 2017
Multiple skill learning	Santucci et al., 2013
Attention Allocation	Di Nocera et al., 2014; Gatsoulis and Mcginnity, 2015

In Reinforcement Learning (RL), an agent learns from experience as it deals with a sequential decision problem. The agent interacts with an “environment” which contains a “critic” that provides the agent with rewards by evaluating the behavior. Through trial-and-error, the agent maximizes the reward over time. With the introduction of intrinsically motivated reinforcement learning (IMRL; Singh et al., 2010), the reward is designed to be a combination of *extrinsic reward* and *intrinsic reward*. While the extrinsic rewards are closely related to the environment itself, the intrinsic reward is used to introduce the effect of factors that are considered to underlie intrinsic motivation. These include novelty, surprise, incongruity etc., which are relative to the agent's learning and memory. With this approach, the intrinsic reward is brought to the fore. Though it may not be directly related to the task the agent is supposed to accomplish, these reward can provide the agent with information that are useful to improve performance (Sorg et al., 2010). This philosophy is in line with the psychological perspectives on intrinsic motivation. Deep neural networks have been used to provide “rich representations” for high-dimensional RL tasks successfully (Mnih et al., 2016). IMs mechanisms have been used as reward functions in Deep Reinforcement Learning (DRL). Intrinsic Motivation in DRL showed improved performance in challenging environments with sparse rewards by providing efficient exploration strategies.

Many of the computational models of IM have organized their structures hierarchically. Typically, a two-level or two-module structure is used. This approach makes it possible to compartmentalize IM from sensorimotor learning. For example, the upper level module generates IM based goals while the lower level explores the environment for reaching particular goal and necessary actions.

In many real-word scenarios, reinforcement learning agents suffer due to the extremely sparse nature of extrinsic rewards. Besides, in an open-ended learning scenario where we would want an agent to learn from the large sensorimotor space by itself. This calls for active learning approaches which can guide an agent by organized and constrained exploration. Intrinsic motivation is used in active learning as a heuristics that helps to maximize learning progress.

Real-world scenarios pose similar problems to artificial agents in many of the other fronts as well. These agents need to be able to plan their motion by finding a path among arbitrary and previously unknown obstacles. Exhaustive searching is computationally expensive- this makes the agents slow, which is quite the opposite of the features we would like to see in a humanoid robot.

Another related trait is an organism's ability to perceive its environment and interact with it. In real world, this would translate to a robot's ability to navigate and using tools in appropriate manner by itself. This resulted in to apply intrinsic motivation to discover object utilities and use them accordingly.

A hallmark of human intelligence is the ability to self-determine goals to achieve particular skills. To be truly autonomous, an artificial system has to discover and select goals on its own. Based on the complexity of these goals, the system may need to decompose it into sub-goals. These sub-goals may not have any tangible rewards at a certain time. Thus, goal discovery and identification from a large space and working toward that by identifying sub-goals become a complex proposition. Intrinsic motivation can be useful in this regard.

While skill acquisition for artificial agents is a complex issue itself, it gets more complicated with the presence of multiple learnable skills. While for humans it is intuitive to choose to learn skills in terms of increasing need and complexity, this is difficult for artificial agents. Closely related to this is the ability to focus attention on a task that is learnable and useful. Computational models of motivation can be used in these cases as well.

The settings listed in Table 2 denote fields which carry significant importance in autonomous mental development. A true autonomous human-like autonomous agent must be able to plan its motions in the continuous space while taking into consideration its own constraints. Throughout its lifetime, it will encounter novel objects and will have to learn about the affordances of objects and how to manipulate them. To acquire competence, it needs to be able to identify goals by itself, learn to compose multiple skills into more complex ones, and allocate attention to learnable situations. As we have predicted, this table provides us with aspects that are fundamental to design machines that help an artificial agent appear to think and act like a human.

#### Mechanism

As we said earlier, in this paper we view the motivation mechanism as a goal generator. It takes the agent's sensations of its world state, and memories of past sensations, as inputs and generates goals that in turn influence action selection. These goals have been expressed in different ways in the literature, ranging from explicit goal structures to implicit utility feedback.

In this section, we describe a number of computational models of intrinsic motivation with respect to the specific nature of the motivation mechanism. A list of possible formal mechanisms of intrinsic motivations was provided in Oudeyer and Kaplan (2007). The list in Table 3 mostly concurs with that typology. The intrinsic reward mechanism works as a part of the organism itself in the reinforcement learning framework. Rewards based on novelty, curiosity and uncertainty are defined with respect to the visited states. Hester and Stone (2015) measured novelty as the distance of the unexplored region of states from the previously visited states. The intrinsic reward is given as proportional to this distance. This motivates the agent to explore the state-actions that are the most different from the ones that are already visited.

**Table 3:** Reward mechanisms in computational models of motivation.

**Mechanism**	**Reference**
Prediction error	Barto et al., 2004; Metzen and Kirchner, 2013
Empowerment	Salge et al., 2014; Mohamed and Rezende, 2015
Learning Progress/Information Gain/KL Divergence	Oudeyer et al., 2007; Baranes and Oudeyer, 2009; Kompella et al., 2012; Frank et al., 2015; Hester and Stone, 2015
Curiosity	Kompella et al., 2012; Di Nocera et al., 2014; Frank et al., 2015; Pathak et al., 2017; Zhelo et al., 2018
Novelty	Metzen and Kirchner, 2013; Gatsoulis and Mcginnity, 2015; Hester and Stone, 2015; Salgado et al., 2016
Surprise	Schembri et al., 2007; Hamann, 2015; Achiam and Sastry, 2017

Using prediction error is inspired by dopamine neurons. In its most basic form, the reward for an event is proportional to the error that was made for a certain event. As agents make prediction of future events based on current ones, this intrinsic reward can enable an agent to focus on an event that has a larger error associated with it. As an agent repeatedly learns more about the event and achieves more success, the intrinsic reward decreases.

Empowerment is a measure of the causal influence an agent has on the perceived world (Klyubin et al., 2005). In computational motivational models, empowerment is typically implemented as a measure of maximizing information or minimizing uncertainty. This provides the agents with adaptability to deal with dynamic environments.

Learning progress or information gain is another highly used reward measure. In this case, the system generates rewards if predictions improve over time, i.e., it tries to minimize prediction errors. By doing this, an agent can focus on states or activities that offer the highest progress. Using learning progress as a reward measure is a robust solution to changing environments. Moreover, learning progress based mechanism result in strategies that autonomously progress from simpler to more complex tasks (Gottlieb et al., 2016).

Curiosity is one of the most widely used measurements of computational intrinsic motivation. In effect, curiosity is defined as a function of learning progress or prediction error. In curiosity-driven exploration, agents are intrinsically rewarded to explore regions which shows higher learnability. Curiosity-driven agents are demonstrated to learn even in situations without any extrinsic rewards (Pathak et al., 2017).

Novelty can be an effective mechanism to implement intrinsic motivation. By encouraging agents to explore states that are highly different than the already visited ones, efficient exploration can be achieved. Measuring novelty typically involves a comparison between the current stimuli and the previous ones. Furthermore, it can also include factors such as habituation, which involves the temporal effects of similar stimuli on novelty.

Surprise is defined as the difference between expectation and outcome. In that case, prediction error can be used as a measurement of surprise and intrinsic rewards. In some other models, surprise is defined as the degree of not expecting an incident.

Some of the implementations have combined the aforementioned reward measures and defined their own (Baranes and Oudeyer, 2010; Sequeira et al., 2011; Hester and Stone, 2015).

#### Function

In this category, we provide a list of functions that result from the implementation of a motivation mechanism in artificial agents. We opted for this aspect with the idea that it would complement the settings that we described previously.

One of the major functions imparted by intrinsic motivation is that of efficient exploration. Agents demonstrate features such as achieving significant states with sparse and delayed rewards, scalability in computationally extensive scenarios. In case of autonomous self-organization, agents could discover their sensorimotor skills by virtue of intrinsic motives without any explicit guidance. It was also shown that computational models of intrinsic motivations foster progressive learning. Intrinsically motivated agents initially spend time in easier situations and then allocate attention to situations with increasing difficulty. This tendency is directly related to the next function of composing complex task by learning simpler tasks first. Artificial agents with intrinsic motivations were significantly faster in completing complex tasks.

The features are listed in Table 4. Various autonomous activities feature prevalently here, with exploratory actions topping the list. This concurs with the very nature and definition of motivated behavior. Intrinsic motivation is supposed to foster exploration and curious behavior that may or may not aid immediate competence and skill acquisition. This demonstrates that existing implementations of computational motivation, irrespective of the settings or reward mechanism, introduces exploratory actions in artificial agents.

**Table 4:** List of functions resulted from computational models of intrinsic motivation.

**Function**	**References**
Efficient Exploration	Frank et al., 2015; Hester and Stone, 2015; Mohamed and Rezende, 2015; Kulkarni et al., 2016; Salgado et al., 2016; Santucci et al., 2016; Achiam and Sastry, 2017; Pathak et al., 2017
Autonomous self-organization	Oudeyer et al., 2007
Progressive learning	Oudeyer et al., 2007; Hester and Stone, 2015
Task composition	Schembri et al., 2007

#### Evaluation Methods

The behavior of an agent comprises the sequences of actions it performs. Evaluating the behavior of an agent driven by computational motivation is not a straightforward task. In case of a motivated agent, we not only want to measure the completion rate for a particular task, but also intend to observe, and measure, the effect of the intrinsic reward on the agent's behavior, organization, and long-term competence and knowledge acquisition. The evaluation methods used in the existing literature are listed in Table 5.

**Table 5:** Methods used to evaluate computational models of intrinsic motivation.

**Evaluation methods**	**References**
Comparison with extrinsic reward	Cameron and Pierce, 1994; Barto et al., 2004; Di Nocera et al., 2014; Hester and Stone, 2015
Goal accomplishment	Kulkarni et al., 2016
Comparison with random and least tried states	Frank et al., 2015
Comparison with greedy approach	Sequeira et al., 2011
Comparing single and multiple intrinsic rewards	Sequeira et al., 2011
Analyzing performance over time	Gatsoulis and Mcginnity, 2015

As a baseline, many of the proposals compare the intrinsically motivated behavior with that of extrinsic reward only. While this largely demonstrates the effectiveness of intrinsic rewards, one may fail to understand the particular influences of intrinsic motivation if it is not extended further. Similar evaluation approaches include comparing intrinsically motivated behavior with random or greedy method. Novel tools to aid the research in computational intrinsic motivation are proposed by groups of researchers from various fields (Natale et al., 2013; Stafford et al., 2013; Taffoni et al., 2013).

### Computational Motivation in Swarm and Multi-agent Settings

While the previous sections examined literature on computational models of motivation, here we focus on the concept of motivation in a social context. This can be a swarm of robots, particles in an optimisation setting, or a multi-agent setting. By providing a summary of the existing works, we point out the fact that in these cases, computational motivation has been used in a limited capacity, almost identical to that of single agent scenarios. We then discuss few of the possible extensions of computational motivation that can be applicable to swarm and multi-agent settings.

Merrick (2015) demonstrated the effects of motive profiles in game-playing agents. With the help of two-player game settings, it was shown that power, achievement, and affiliation motives can lead to various emergent behaviors. Moreover, in case of evolutionary algorithm for creating motivated agents, it was shown that motivated agents were more diverse and achieve higher incentive than their non-motivated versions. Evolutionary models of intrinsically motivated agents were simulated in a multi-agent setting in by Shafi et al. (2012). A static incentive model was used to generate agents that change their motives over time.

Hardhienata et al. (2012, 2014a,b) incorporate achievement, power, affiliation and leadership motivation in a Particle Swarm Optimization (PSO) setting. The application area the agents are tested in is task allocation. Incentives are defined as distance of the agent from the task and the number of agents around the task. The incentive value, along with motive profiles, help inform an agent which task and neighborhood to choose. Their work shows the effect of motivational profiles on an established algorithm such as PSO and task allocation.

Klyne and Merrick (2016) used computational models of motivation to generate dynamic fitness functions for PSO. The convergence of the swarm on the generated fitness function is tested in a workplace hazard mitigation scenario. The two approaches to model motivation in this framework were novelty and curiosity. Curiosity was implemented via K-Means neural network. The results showed this approach is more applicable for decentralized data sources. In case of a centralized source, background subtraction was used. This is an image processing technique to detect novel objects in successive image frames. Evaluation metrics include ability of goal generation and convergence of swarm once the goal is generated. In some cases, the generated fitness function has too many local maxima for the swarm to converge on the right place.

Saunders and Gero (2004) used flocking and a social force model to design curious agents. These agents perform evaluations of environments which are designed to prompt exploration, e.g., art galleries. Interestingness is modeled using the Wundt curve: the most interesting situations are the ones that have moderate novelty. Situations are measured with hedonistic values and agents will move toward stimuli having higher value. Their results showed that these curious agents spend significantly more time in environments which were designed “better.” Linkola et al. (2016) used novelty to create a group of creative and curious agents. A society of homogenous agents is created, with each one having an individual memory. The behavior of the population is modeled via iterations. In each iteration, each agent creates a candidate artifact bases on the current location and memory. The agents then collectively decide which of the candidate artifacts can be added the repository. The agents are self-critical and have veto power. As the results show, self-criticism lowers the amount of collaborative effort in evaluating candidate artifacts while veto power increases novelty. Galvao et al. (2015) implement the notion of novelty search (Lehman and Stanley, 2011) in PSO.

Table 6 summarizes the existing work that incorporates motivation in multi-agent and swarm settings using the headings of setting, mechanism, and function introduced in the previous section. As the settings column demonstrates, most of the works have been implemented in PSO and game-theoretic settings. Moreover, in many of these works the focus was on the function rather than the motivation mechanism. As a result, they lack a detailed analysis of the implications of motivation being implemented in a social context. This is where our proposals of the newer settings and possibilities come in.

**Table 6:** A summary of the work using motivation in social context in terms of their setting, function and mechanism.

**Setting**	**Function**	**Mechanism**	**Reference**
Various Games	Game theoretic analysis	Motive Profiles	Merrick, 2015
Prisoner's Dilemma	Game theoretic analysis	Motive Profiles	Shafi et al., 2012
PSO	Task Allocation	Motive Profiles	Hardhienata et al., 2012, 2014a,b
PSO	Generating dynamic fitness function	Curiosity Novelty	Klyne and Merrick, 2016
Flocking, Social Force Model	Design Evaluations	Curiosity	Saunders and Gero, 2004
Multi-agent systems	Generating Creative Artifacts	Novelty	Linkola et al., 2016
PSO	Grammatical Swarm	Novelty	Galvao et al., 2015

**Figure 2:**
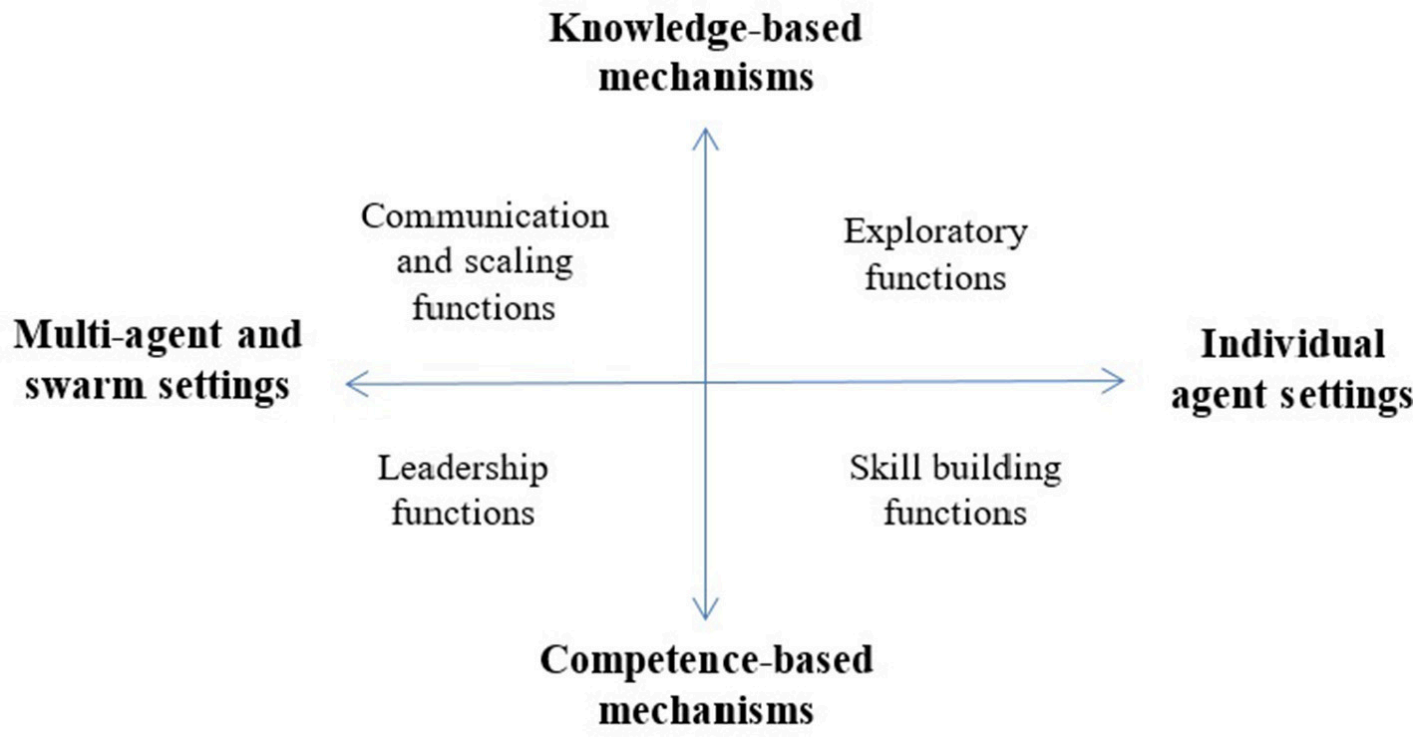
Expanding the view of settings for computational models of motivation also expands possible functions.

Figure 2 hypothesizes about the new functions that we may achieve in motivated agents if augment the field with new multi-agent and swarm settings. The two extremes of the vertical axis in Figure 2 represent the knowledge-based and competence-based mechanisms of intrinsic motivation. As we pointed out earlier, this is the most general categorization of the computational models, which covers a range of detailed mechanisms discussed in Section Mechanism. In the upper part of the vertical axis, we have the knowledge-based models which produce exploratory functions and acquire knowledge about the surrounding environment or the world. In the lower part, we have the competence-based models which function to improve skills. On the other hand, the horizontal axis represents an expanded view of possible e agent settings. On the right-hand side, we have individual agent settings. On the left side, we introduce multi-agent and swarm settings. We hypothesize that these settings will see motivated agents performing new functions including leadership, for example leading agents that do not have intrinsic motives, and scaling and communication functions. By this we mean that extension to the multi-agent settings permits intrinsically motivated agents to scale to problems that cannot be solved by a single agent.

In addition to enabling new functions, we hypothesize a range of ways that motivation may be embedded in multi agent or swarm settings. For example, motivation may be distributed among multiple agents or it may be shared. In the first case, the motivation mechanism of each agent is processed and acted upon by itself. The group behavior rules, such as flocking, will still affect the emergent behavior. In case of shared motivation, agents will interact with each other while constructing the motivated behavior. The underlying assumption in this case is that the interpersonal factors, such as aligning one's goal to a friend's interests, will play an important factor to determine the group behavior. With the introduction of this concept, we can explore notions such as conformity, divergence and living up to the expectations of others. These concepts have been investigated in human psychology (Fishbach et al., 2016) but not yet in the computational motivation research.

**Figure 3:**
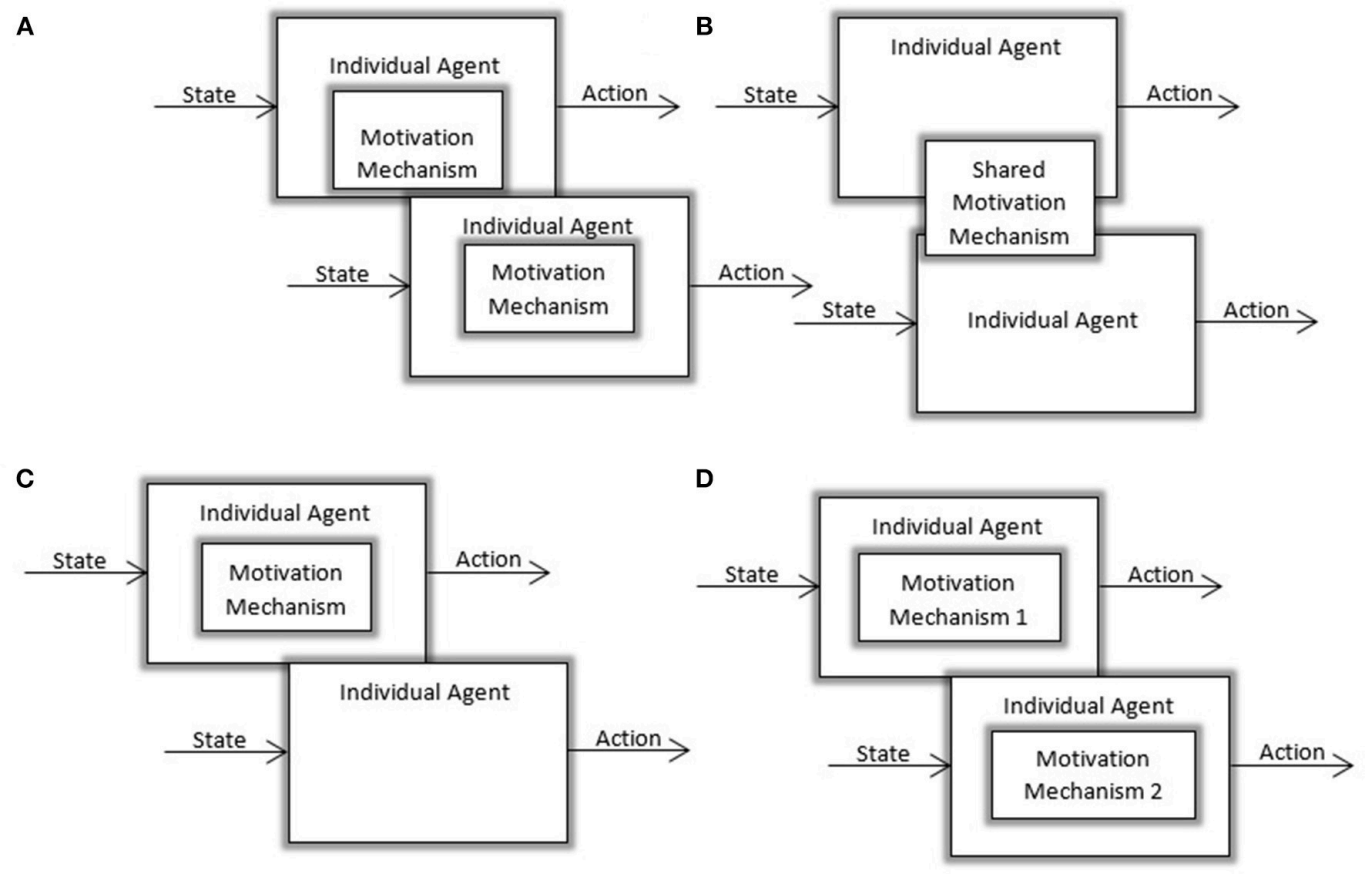
Visualization of different possible multi-agent settings: **(A)** Multi-agent setting with homogeneous agents and decentralized motivation mechanisms **(B)** Multi-agent settings with homogeneous agents using a centralized motivation mechanism **(C)** Heterogeneous society with a subset of motivated agents **(D)** Heterogeneous society with agents using different motivation mechanisms.

Moreover, all agents in a group may have the same motives or they may have different motives. While some agents in a group can be motivated to improve personal knowledge and competence, others can pursue that knowledge for gaining control over the group. This can result in homogeneous and heterogeneous groups of motivated agents. Some of the existing works investigated the effects of different motives but only a few looked into heterogeneously motivated artificial agents coexisting as a group. Likewise, all agents may be motivated agents or only a subset of agents may be motivated, and others may not possess such models. These variants are illustrated in Figure 3.

## Discussion

In this section, we discuss the challenges associated with computational modeling of intrinsic motivation in social context and conclude the paper.

### Research Challenges

There are quite a few challenges that emerge as we ponder the notion of multiple or swarms of agents equipped with computational motivation. We discuss these challenges from the four aspects we had described in section Structuring Existing Approaches to Computational Motivation- settings, rewards, functions, and evaluation. Combining these aspects with the possible extensions discussed in section Computational Motivation in Swarm and Multi-Agent Settings, we present a set of research challenges. These research challenges encapsulates our discussion in the previous sections and provide an overview of the future research involving computational motivation in a social context.

#### Settings to Accommodate the Social Context

Most of the existing works on computational motivation have used PSO or flocking as setting. Though they provide a structured base to investigate motivation, they are too limited in many ways. Human and animal motivation mechanisms involves interaction patterns that are significantly more complex than these restricted settings. We feel eventually there will be a need to generate more flexible, complex, and accommodating multi-agent settings. Settings such as Belief-Desire-Intention (BDI) architecture (Rao and Georgeff, 1991), game theory (Parsons and Wooldridge, 2002), multi-agent reinforcement learning (Busoniu et al., 2008), and swarm intelligence (Brambilla et al., 2013) can be useful in this context. Researchers from these respective areas will have to identify the existing properties and extend the architecture to accommodate computational models of motivation.

As the notion of multiple motivated agents is introduced, quite a few features for these settings can be proposed. These were not applicable to single agents, but pertinent in multi-agent scenarios. As discussed previously, we might have a homogenous set of agents equipped with the same motivation mechanism. Many of the current works are exploring this line of work where each agent or particle in a swarm has the same motivation. However, there can be a heterogeneous setting where the agents can be motivated through varying mechanisms of motivation. For example, one agent can act as an informed individual and the other members of the group can be following that agent for achieving a goal or skill. Though there are current works that propose and measure the effectiveness of various motive profiles, there is no study that focuses on a swarm of agents that are equipped with different forms of computational motivation. Another related open area of research will consist of scenarios that will be able to accommodate a temporal change of the motivation mechanism. Imagine a scenario where agents start with having affiliation motive as the primary driving factor but changes to power motives after gaining certain knowledge or skillset.

#### Novel Mechanisms and Rewards

The current work on the computational models of motivation is largely comprised of translating the psychological theories of motivation into mathematical and computational models. Motivation can have many facets and dimensions when it is compounded by social factors such as presence of and interaction with other individuals. There are some works on various motive profiles, as we discussed in the earlier sections. However, the social theories of motivation are yet to be generally implemented as computational models.

Psychological studies in organizational behavior, student motivation and performance reveal interesting facts about the mechanism of intrinsic motivation in social contexts. It has been observed that support for competence, relatedness and autonomy helps increase children's intrinsic motivation (Ryan and Deci, 2000b). In an educational environment involving high-school students, the authors showed that optimal challenge and performance feedback facilitates competence while relatedness is increased by meaningful parental involvement and peer acceptance (Dörner and Güss, 2013). If we want to model motivation in a social context, we would need to utilize the psychological studies and introduce novel reward mechanisms such as relatedness, feedback and autonomy support.

The next generation of computational models would need to implement, and in many ways, extend and augment the psychological theories. Motivated artificial agents can provide both psychologists and computational intelligence researchers with an avenue of proposing and evaluating various psychological theories. A swarm of motivated agents in a simulated environment can be used to model and predict motivational tendencies such as curiosity and novelty. This can be an alternative to questionnaire-based analysis that typically takes place in psychological studies.

#### Functions

The existing works have shown the inception of various emergent functional behavior as a result of computational motivation mechanism. With multiple agents, the emergent functions are more versatile, complex and significant. This is due to the fact that within a social context the agents are interacting not only with the environment but also within themselves. Factors such as shared goals and conflicting motivations can lead to interesting emergent behaviors in swarms and multi-agent systems. Seemingly simple and primitive rules can be combined to produce complex behavior patterns. One challenge of the future research would be to devise mechanism to generate such behaviors from the primitive rules and through motivation mechanisms. For example, one can think of a flock of agents swarming through an environment by virtue of the intrinsic motivation values. In this case, we will need to design, estimate, and adapt the effects of motivational mechanisms on individual agent as well as on the group behavior. While it would have been relatively simpler to achieve this in an individual agent setting, it would be much more complex in case of multiple interacting agents. It will be interesting and useful to observe the effect of different motivation mechanisms on this mapping between primitive rules and emergent behavior.

#### Evaluating Behaviors

As we have pointed out already, evaluating the consequences of implementing motivation (especially intrinsic motivation) is not quite straightforward. The state-of-the-art single agent architectures still suffer from a lack of widely accepted behavior metrics capable of measuring the effects of computational motivation. With multiple motivated agents, the challenge of measuring emergent motivated behavior becomes increasingly complex. It would be a challenge to determine behavior metrics that can embody the motivated behavior of multiple interacting agents.

We emphasize on the evaluation as it has significant implications involving the motivation mechanism and expected behavior. A causal relation between the motivation mechanism and behavior can be used to generate artificial agents with intended motive profiles and tune them as need be. The current proposals do not establish a rigorous or mathematical relation between the motivation parameters (e.g., reward measures) and the corresponding behavior (e.g., skills acquired). As more complex scenarios involving multiple agents are introduced, this becomes even more challenging. If the next generation of these proposals can determine the relation between the controlling parameters and emergent behavior more rigorously, we will be able to derive more applications of the motivated agents. Determining appropriate behavior metrics will play a significant part in this regard.

## Conclusion

Motivated artificial agents are designed to acquire knowledge and skills in an open-ended setting. These features can provide new horizons in artificial intelligence, machine learning and computational intelligence in general. In this survey, we have summarized the implementations of motivation in artificial agents. We provided definitions, background, and state-of-the-art of the field of computational motivation. We have provided a new typology through which the current research can be categorized through four main aspects: setting, mechanism, function, and evaluation method. Through a systematic discussion, we demonstrated the fact that there is limited work using computational motivation in multi-agent and swarm settings. Following a detailed discussion on this topic, we presented the major research challenges for achieving societies of multiple motivated agents. We believe our contribution in this paper will help researchers to further identify and explore these open research topics.

## Author Contributions

MK and KK conceived the idea of the paper. MK conducted the analysis and wrote the first draft. KK and MB provided critical feedback and suggestions to organize the subsequent revisions. KK contributed several diagrams and related texts. KK and MB overviewed the whole paper writing process. MK finalized the paper.

### Conflict of Interest Statement

The authors declare that the research was conducted in the absence of any commercial or financial relationships that could be construed as a potential conflict of interest.
